# Integrative modeling of malignant epithelial programs in EGFR-mutant LUAD via single-cell transcriptomics and multi-algorithm machine learning

**DOI:** 10.3389/fimmu.2025.1661679

**Published:** 2025-10-14

**Authors:** Weiran Zhang, Lin Tan, Qiuqiao Mu, Han Zhang, Daqiang Sun

**Affiliations:** ^1^ Tianjin Chest Hospital, Tianjin University, Tianjin, China; ^2^ Qingdao Hospital, University of Health and Rehabilitation Sciences (Qingdao Municipal Hospital), Qingdao, China

**Keywords:** LUAD, scRNA-seq, EGFR, machine learning, immunotherapy, PERP

## Abstract

**Background:**

Lung adenocarcinoma (LUAD) is the most common subtype of non-small cell lung cancer, with EGFR mutations serving as key oncogenic drivers. However, patients harboring EGFR mutations exhibit considerable heterogeneity in clinical outcomes and treatment responses. Characterizing the malignant features of EGFR-mutant epithelial cells may facilitate improved stratification and personalized therapeutic strategies.

**Methods:**

Using publicly available single-cell RNA sequencing data, malignant epithelial cells were identified in EGFR-mutant LUAD samples via inferCNV and k-means clustering. Pseudotime trajectories were constructed using Monocle2, and branch-specific genes were extracted for functional analysis. Differentially expressed genes were integrated with TCGA bulk transcriptomic data, and ten machine learning algorithms were applied to construct the EGFR Mutation-Associated Malignant Epithelial Cell-Related Signature (EGFRmERS). The prognostic value of EGFRmERS was validated across multiple independent cohorts. Associations between EGFRmERS and immune infiltration, immunotherapy response, tumor mutation burden (TMB), and copy number variations (CNVs) were systematically assessed. The performance of EGFRmERS was also benchmarked against previously published LUAD prognostic signatures. Finally, the core gene PERP was selected for *in vitro* functional validation, including qRT-PCR, Transwell migration/invasion, and colony formation assays.

**Results:**

EGFR-mutant epithelial cells were classified into subclusters with varying malignant potential, enriched in pathways such as cell cycle regulation and DNA repair. The EGFRmERS signature robustly predicted patient prognosis across multiple cohorts and outperformed existing models. High EGFRmERS scores were associated with an immunosuppressive microenvironment, reduced immunotherapy responsiveness (as indicated by TIDE and IPS scores), elevated TMB, and increased genomic instability. PERP was identified as a key gene, highly expressed in LUAD and associated with poor prognosis. Functional assays confirmed its role in promoting cell migration, invasion, and clonogenic capacity.

**Conclusions:**

This study delineates key malignant programs in EGFR-mutant epithelial cells at the single-cell level and proposes a robust prognostic scoring system, EGFRmERS, with strong predictive power for survival and immunotherapy benefit. PERP was identified as a potential therapeutic target, offering novel insights for precision stratification and treatment in EGFR-mutant LUAD.

## 1 Introduction

LUAD is one of the most common and deadly malignancies worldwide, representing the major histological subtype of non-small cell lung cancer (NSCLC) (1). With advances in molecular classification, mutations in the epidermal growth factor receptor (EGFR) have been identified as the most frequent oncogenic driver in LUAD, particularly prevalent in Asian populations where the mutation rate exceeds 40% (2–4). Although EGFR-targeted tyrosine kinase inhibitors (TKIs) have achieved significant therapeutic success, a proportion of patients still experience disease progression or develop resistance during treatment, indicating substantial biological heterogeneity within the EGFR-mutant subgroup (5–7). Moreover, EGFR-mutant LUAD typically exhibits a “cold” immune phenotype and shows limited response to immune checkpoint inhibitors (ICIs), suggesting that this subtype possesses distinct features in terms of tumor microenvironment (TME), immune escape mechanisms, and gene expression profiles (8, 9). Therefore, EGFR mutation status alone may be insufficient to predict prognosis or therapeutic outcomes, highlighting the urgent need for more refined stratification strategies to improve personalized treatment approaches.

In recent years, numerous prognostic models have been proposed for LUAD, aiming to stratify patients based on gene expression, mutation burden, immune profiles, and other factors (10, 11). However, most of these models rely on bulk RNA sequencing data, which cannot capture the cellular heterogeneity within tumors or reflect the functional differences among tumor cells. The emergence of single-cell RNA sequencing (scRNA-seq) technology has enabled researchers to explore the transcriptional states and molecular features of distinct cell populations at single-cell resolution (12, 13). Previous studies have shown that the transcriptional characteristics of epithelial cells not only represent the biological behavior of tumors but also influence immune cell infiltration and response to immunotherapy (14, 15). Nevertheless, there is still a lack of studies focusing specifically on the malignant epithelial cells in EGFR-mutant LUAD, which limits the development of more specific prognostic models and therapeutic prediction tools derived from the tumor itself.

At the same time, machine learning has been increasingly applied in biomedical research, offering advantages such as precise feature selection, robustness in high-dimensional data, and the ability to integrate multiple variables to construct predictive models (16, 17). Compared to traditional univariate or linear models, machine learning algorithms can capture complex nonlinear relationships and identify critical feature combinations, thereby improving the predictive power and generalizability of prognostic tools. Integrating malignant epithelial cell features identified at the single-cell level with multiple machine learning approaches holds great promise for building individualized risk assessment systems with clinical relevance.

In this study, we focused on malignant epithelial cell subsets from single-cell transcriptomic data of EGFR-mutant LUAD. By performing copy number variation analysis and pseudotime trajectory reconstruction, we identified key differentiation features within this population. We then integrated bulk RNA-seq data from the TCGA-LUAD cohort and multiple external validation datasets to construct and validate the EGFR mutation malignant epithelial cell-related signature (EGFRmERS) using ten machine learning algorithms. Finally, we evaluated the biological significance and clinical relevance of this signature through comprehensive analysis of immune characteristics, drug sensitivity profiles, and *in vitro* functional experiments. This study provides a novel perspective for risk stratification and immunotherapy precision in patients with EGFR-mutant LUAD.

## 2 Method

### 2.1 Multi-omics data acquisition and integration

ScRNA-seq data were obtained from the GEO database (accession number: GSE171145), comprising nine tumor samples from eight treatment-naïve LUAD patients. Among them, four samples harbored EGFR mutations, while five were EGFR wild-type (3). Detailed information on the EGFR mutation status for each patient can be found in **Supplementary Table 1**. The dataset includes comprehensive clinical annotations and EGFR mutation information. Bulk RNA-seq data from the TCGA-LUAD cohort were downloaded using the TCGAbiolinks R package (18), including transcriptomic profiles, somatic mutation data, and clinical annotations. Additionally, six independent NSCLC cohorts were retrieved from GEO as external validation datasets, as detailed in the Results section. To mitigate potential batch effects among different datasets, we applied the ComBat function from the sva R package for batch correction (19), ensuring comparability and robustness in downstream analyses. We further utilized multiple bioinformatics tools and platforms to support functional and immunological investigations, including GISTIC2.0 (20) for copy number variation analysis, TIMER2.0 for immune cell infiltration estimation (21), TIDE for immunotherapy response prediction, and SubMap (22) for immunophenotype mapping.

### 2.2 Quality control and clustering of scRNA-seq data

The scRNA-seq data were processed using the Seurat package (version 4.4.0). Initially, a Seurat object was created from the raw count matrix, followed by quality control to exclude low-quality or ambiguous cells. Filtering criteria included: number of detected genes (nFeature) between 500 and 10,000, total UMI counts (nCount) between 1,000 and 100,000, mitochondrial gene percentage below 40%, and hemoglobin gene percentage below 5%. Following normalization (NormalizeData) and identification of highly variable genes (FindVariableFeatures), dimensionality reduction was performed using principal component analysis (PCA). To minimize batch-specific variations between samples, we applied the Harmony algorithm for batch effect correction using orig.ident as the grouping variable (group.by.vars), while keeping all other parameters at their default settings. The top principal components were used as input for integration, and convergence plots were generated to monitor the optimization process. Batch correction effectiveness was evaluated by visual inspection of PCA and UMAP projections before and after correction. Cell clustering was then performed using a shared nearest neighbor (SNN) graph-based approach with the resolution parameter set to 1.0. Clustering results were visualized via UMAP (Uniform Manifold Approximation and Projection) for two-dimensional projection. Cell type identification was guided by canonical marker gene expression and further assisted by reference-based methods such as SingleR and scType. To enhance the visualization of gene expression patterns, we employed tools such as Nebulosa (23) (for density-based feature plots), SCP (24) (for plot layout organization), and plot1cell (for high-quality rendering of single-cell results).

### 2.3 Differential cell–cell communication analysis between EGFR-mutant and wild-type LUAD

To investigate how EGFR mutation status affects intercellular signaling within the tumor microenvironment, we performed cell–cell communication analysis using the CellChat R package (25). Expression data and cell-type annotations were extracted from the processed single-cell dataset for both the EGFR-mutant (Mut) and wild-type (WT) groups. Potential ligand–receptor interactions were inferred based on a curated signaling database focused on secreted signaling pathways. Communication probability between cell types was then estimated to construct interaction networks for each group. The number and strength of signaling events were compared between groups, and the signaling roles of different cell types—such as senders or receivers—were assessed. Signaling pathways that showed increased or decreased activity in the EGFR-mutant group were identified and visualized using network and heatmap plots to highlight specific source–target cell pairs. All analyses were conducted using the default settings and standard workflow recommended by the CellChat framework.

### 2.4 Clustering analysis and identification of malignant epithelial cells

To identify malignant epithelial subpopulations, we performed unsupervised clustering based on the CNV expression matrix inferred by inferCNV (26). Endothelial cells were used as the reference group to normalize CNV signals. All epithelial cells were then clustered using the k-means algorithm, with the number of clusters set to five. The CNV profiles of each cluster were assessed through heatmap visualization and CNV score distribution. Clusters 1, 3, 4, and 5 exhibited markedly elevated CNV levels and substantial deviations from reference cells, and were thus defined as malignant epithelial populations. These identified subclusters were used in downstream analyses, including dimensionality reduction, functional enrichment, and assessment of intratumoral heterogeneity.

### 2.5 Reconstruction of malignant epithelial cell differentiation trajectory

To explore the dynamic progression and transcriptional heterogeneity of malignant epithelial cells, pseudotime analysis was performed using the Monocle2 package (27). A CellDataSet object was constructed using raw count data, with size factors and dispersions estimated for normalization and variance modeling. Lowly expressed genes were filtered out, and the top 2000 differentially expressed genes across epithelial subclusters were selected for ordering. Dimensionality reduction was conducted using the DDRTree algorithm to infer the developmental trajectory and project cells along a continuous pseudotime axis. Each cell was assigned a pseudotime value and a discrete state. Visualization of the trajectory was performed by coloring cells based on cluster identity, pseudotime, and inferred cell states. To investigate gene expression changes associated with lineage bifurcation, BEAM analysis was applied at the primary branching point. Genes showing branch-dependent expression patterns were identified, and clustering was performed to group genes with similar expression dynamics. Functional enrichment analysis of each gene module was conducted using the ClusterGVis framework, and results were visualized as annotated heatmaps incorporating both expression patterns and biological process annotations.

### 2.6 High-dimensional weighted gene co-expression network reveals functional modules in malignant epithelial cells

To identify co-expressed gene modules in malignant epithelial cells, a high-dimensional weighted gene co-expression network analysis (hdWGCNA) was conducted (28). The analysis began with the extraction of gene expression data from malignant epithelial subsets, followed by the exclusion of lowly expressed genes and the selection of highly variable genes for downstream analysis. A weighted correlation matrix was constructed, and a suitable soft threshold was determined to ensure the network conformed to a scale-free topology. Hierarchical clustering was then applied to group genes with similar expression patterns. Dynamic tree cutting was used to define distinct co-expression modules, with each module assigned a unique color label. Module eigengenes (MEs), representing the principal components of each module, were calculated to reflect the overall expression profile of the corresponding module. These eigengene scores were subsequently projected onto a two-dimensional embedding to visualize the spatial distribution of modules across different malignant cell subsets. For each module, the top ten genes showing the strongest correlation with the module eigengene were identified as representative hub genes. To further investigate module-specific expression patterns, violin plots were generated to compare module scores across distinct malignant subclusters. Additionally, the relationships among different modules were evaluated by constructing a correlation matrix based on their eigengenes, providing insights into potential co-regulation or functional interactions between modules.

### 2.7 Machine learning-based generation of the EGFRmERS score for LUAD prognosis

To develop a multi-gene prognostic scoring system, the TCGA-LUAD cohort was used as the training dataset, and a set of candidate genes was subjected to feature selection and model development. In this process, we chose the intersection of three gene sets: hdWGCNA module genes, TCGA differentially expressed genes, and marker genes from prognostic subclusters. The primary goal of this strategy was to integrate data from different sources and select genes that are highly related to clinical prognosis, thereby improving the specificity of the model. However, this approach may lead to the filtering out of potentially important genes, especially in cases of functional overlap between genes. To ensure the reliability of the model, we validated the selected genes across multiple independent datasets and used cross-validation methods to ensure consistency and stability of the selected genes in different datasets. Ten widely used machine learning algorithms were employed, including stepwise Cox regression, Lasso, Ridge, partial least squares regression for Cox (plsRcox), CoxBoost, random survival forest (RSF), generalized boosted regression modeling (GBM), elastic net (Enet), supervised principal components (SuperPC), and survival support vector machine (survival-SVM). Each method was trained under a consistent cross-validation framework to evaluate its predictive performance. The resulting models were further validated across multiple independent external cohorts to assess generalizability and robustness, providing a basis for selecting the optimal prognostic model.

To comprehensively assess the robustness and generalizability of the EGFRmERS signature, we conducted external validations across six independent GEO cohorts (GSE31210, GSE50081, GSE30219, GSE37745, GSE26939, and GSE42127) in addition to the TCGA-LUAD dataset. For each dataset, the EGFRmERS score was calculated using the final RSF+SuperPC model. Patients were stratified into high- and low-risk groups based on the median score within each cohort. Kaplan–Meier survival curves and log-rank tests were applied to compare overall survival between groups. Predictive accuracy was further assessed using time-dependent ROC curves at 1-, 3-, and 5-year intervals, with AUC values calculated accordingly.

To benchmark the prognostic performance of EGFRmERS, we computed the concordance index (C-index) and compared it with conventional clinical variables (age, gender, stage) in each dataset. Additionally, EGFRmERS was compared against previously published LUAD prognostic gene signatures using the same C-index metric. These comparisons were visualized to highlight the superiority or complementarity of EGFRmERS across multiple datasets and evaluation criteria.

For survival analyses, model-related comparisons (e.g., EGFRmERS) used median-based stratification within each cohort, whereas subcluster-specific signature analyses (**Supplementary Figures 2A–E**) used the optimal cut-off determined by survminer::surv_cutpoint.

### 2.8 Comprehensive evaluation of drug sensitivity and immunotherapy response based on EGFRmERS

To evaluate the potential therapeutic implications of the EGFRmERS signature, we conducted a series of analyses related to drug sensitivity and immunotherapy response. Drug sensitivity prediction was performed using data from the CTRP and PRISM pharmacogenomic databases (29), which contain compound response profiles across a wide range of cancer cell lines. Based on gene expression data, the estimated area under the dose–response curve (AUC) values were compared between high- and low-EGFRmERS subgroups to assess potential differences in drug responsiveness. In addition, Spearman correlation analysis was applied to evaluate the relationship between EGFRmERS scores and AUC values across drugs.

For immunotherapy prediction, we examined the expression levels of immune checkpoint-related genes in different EGFRmERS groups and assessed their correlations with the signature. To further predict immunotherapy efficacy, immune-related scores including TIDE (Tumor Immune Dysfunction and Exclusion) (30), IPS (Immunophenoscore), and Exclusion scores were compared between groups. SubMap analysis was also performed to predict the likelihood of response to anti–PD-1 or anti–CTLA-4 therapies based on transcriptomic similarities with known responder profiles.

### 2.9 Evaluation of immune infiltration and stromal features based on EGFRmERS stratification

To explore the relationship between EGFRmERS scores and the tumor immune microenvironment, we retrieved immune cell infiltration data generated by seven widely used algorithms (TIMER, CIBERSORT, CIBERSORT-ABS, QUANTISEQ, MCPCOUNTER, XCELL, and EPIC) from the TIMER2.0 database. A comprehensive immune heatmap was constructed to visualize the compositional differences between high- and low-score groups. Additionally, the ESTIMATE algorithm was applied to calculate stromal scores, immune scores, and ESTIMATE composite scores for each sample, thereby assessing the association between EGFRmERS scores and tumor purity. Moreover, a set of immunomodulatory genes—including co-stimulatory molecules, co-inhibitory molecules, and antigen presentation-related genes—was selected to comprehensively characterize their multi-omics features across EGFRmERS subgroups. This analysis integrated mRNA expression, DNA methylation levels, Spearman correlations between expression and methylation, and copy number variation (CNV) data derived from the TCGA-LUAD cohort. The immunomodulator gene list was curated from the literature, aiming to provide a systematic view of immune regulation heterogeneity under different EGFRmERS conditions.

### 2.10 Association of EGFRmERS with tumor mutational burden and mutation landscape

The mutation annotation format (MAF) file for the TCGA-LUAD cohort was downloaded and analyzed using the maftools package (31) to evaluate tumor mutation burden (TMB) and visualize the mutational landscape. TMB was defined as the number of non-synonymous mutations per megabase. Based on the median EGFRmERS score, patients were stratified into high- and low-risk groups. Differences in TMB between the groups were assessed, and the correlation between TMB and EGFRmERS score was calculated. Furthermore, combined survival analysis was performed using the survminer and survival packages to investigate the prognostic significance of TMB in conjunction with the EGFRmERS score. To assess genomic instability between EGFRmERS-defined subgroups, copy number variation (CNV) data generated by GISTIC2.0 were retrieved from the GDC portal. Amplification and deletion events at the segment level were extracted and visualized across chromosomes. CNV distribution plots were generated for high- and low-EGFRmERS groups to compare genome-wide chromosomal instability patterns.

### 2.11 Cell culture and transfection

Human lung adenocarcinoma cell lines A549 and H1299 were cultured in RPMI-1640 medium supplemented with 10% fetal bovine serum and 1% penicillin–streptomycin, and maintained at 37°C in a humidified incubator with 5% CO_2_. Cells in the logarithmic growth phase were used for subsequent experiments.

Transfection of siRNAs targeting PERP was performed using Lipofectamine™ 3000 reagent according to the manufacturer’s instructions. siRNAs were mixed with transfection reagent in serum-free Opti-MEM medium to form complexes, which were then added to the plated cells. After 48 hours of transfection, cells were harvested for downstream assays. A non-targeting siRNA (siNC) was used as the negative control.

### 2.12 Quantitative real-time PCR

Cells were washed with PBS and lysed using TRIzol reagent to extract total RNA. RNA concentration and purity were measured using a NanoDrop 2000 spectrophotometer. Equal amounts of RNA were reverse-transcribed into cDNA following the manufacturer’s instructions. qRT-PCR was performed using SYBR Green dye on a real-time PCR system. Each 20 µL reaction mixture contained cDNA template, forward and reverse primers, and SYBR Green master mix. The amplification protocol consisted of an initial denaturation at 95°C for 30 seconds, followed by 40 cycles of 95°C for 5 seconds and 60°C for 30 seconds. Each sample was analyzed in triplicate. Relative gene expression levels were calculated using the 2^−ΔΔCt method, with GAPDH as the internal control. Primer sequences are listed in **Supplementary Table 2**.

### 2.13 Transwell migration and invasion assays

Transwell chambers with 8 μm pore membranes were used to assess cell migratory and invasive abilities. For migration assays, cells were suspended in serum-free medium and seeded into the upper chamber (5×10^4^cells per well). The lower chamber was filled with complete medium containing 10% fetal bovine serum as a chemoattractant. For invasion assays, the upper chamber membrane was pre-coated with diluted Matrigel, and the remaining steps were identical to the migration assay. After incubation for 24–48 hours at 37°C with 5% CO_2_, non-migrated or non-invaded cells on the upper surface were removed. Cells on the lower surface were fixed and stained with 0.1% crystal violet. Five random fields were selected for cell counting, and images were analyzed using ImageJ software.

### 2.14 Colony formation assay

Treated cells were seeded into 6-well plates at a low density (500–1000 cells per well) and cultured for 10–14 days until visible colonies appeared. Cells were gently washed twice with PBS, fixed in 4% paraformaldehyde for 15–20 minutes, and then stained with 0.1% crystal violet for 30 minutes. After removing excess dye and rinsing with water, the plates were imaged, and colonies larger than 1 mm in diameter were counted using ImageJ software to assess clonogenic potential.

### 2.15 Statistical analysis

All statistical analyses were performed using R software (version 4.2.0) and GraphPad Prism (version 9.0). Comparisons between two groups were conducted using the Wilcoxon rank-sum test or Student’s t-test, depending on data distribution. For multiple group comparisons, the Kruskal–Wallis test or one-way ANOVA was applied. Survival differences were assessed by the Kaplan–Meier method with log-rank test. Correlation analyses were conducted using Spearman or Pearson correlation as appropriate. Nomogram construction and decision curve analysis were conducted using the “rms” and “rmda” packages in R. A p-value < 0.05 was considered statistically significant unless otherwise specified.

## 3 Results

### 3.1 Construction of a single-cell atlas and annotation of major cell types in LUAD

**Figure 1:**
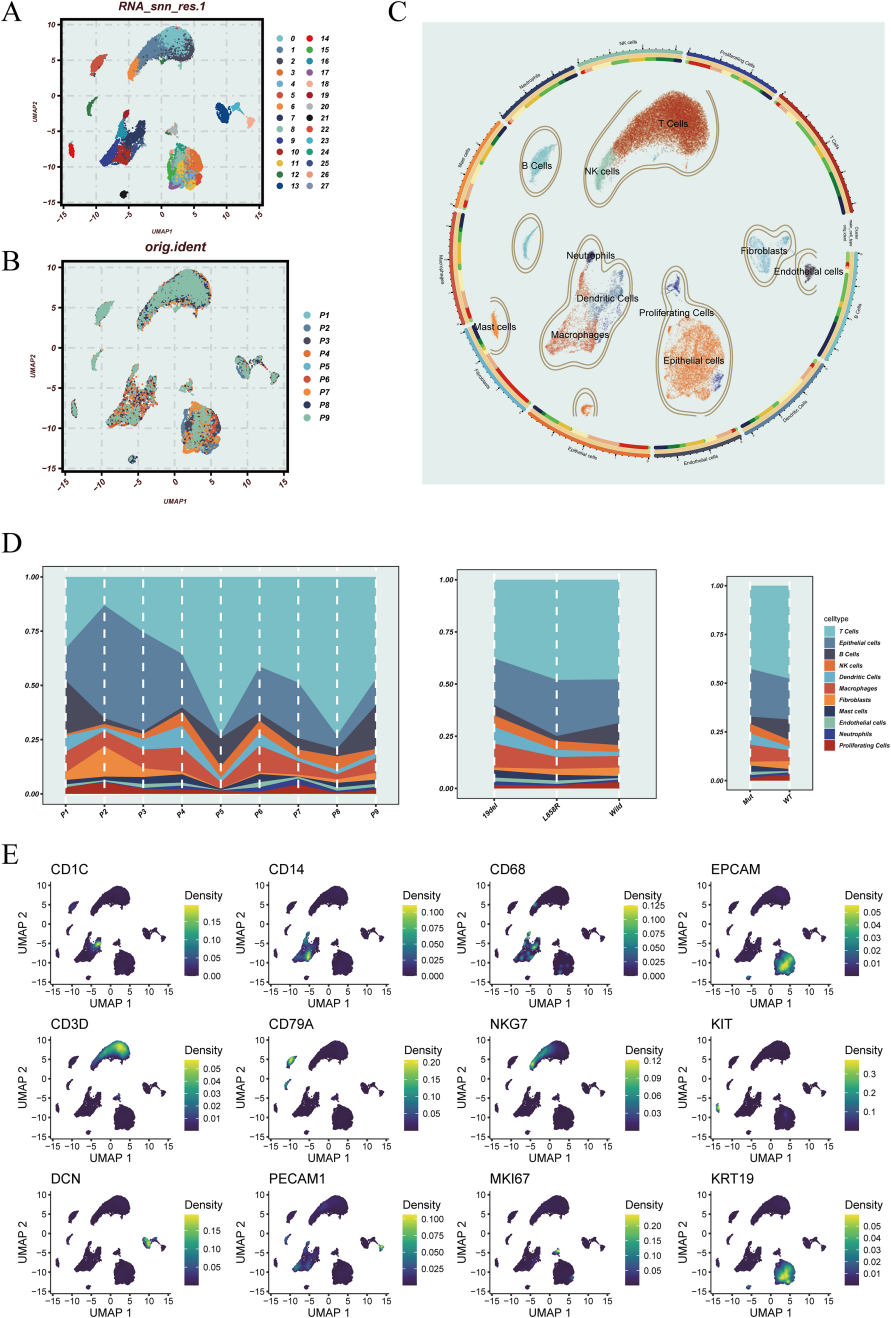
Dimensionality reduction, sample distribution, cell annotation, and cell-type characteristics of the single-cell transcriptomic data. **(A)** UMAP plot showing cell clusters identified using PCA and the shared nearest neighbor (SNN) graph-based clustering algorithm. **(B)** UMAP visualization of the original sample origin for each cell. **(C)** Cell type annotation based on canonical marker genes, identifying major lineages including T cells, B cells, epithelial cells, dendritic cells, macrophages, and others. **(D)** Proportional distribution of annotated cell types across individual patients (left), different EGFR mutation subtypes (middle), and EGFR mutation status (right). **(E)** UMAP feature plots displaying the expression of representative marker genes, including CD1C, CD14, and CD68; EPCAM and KRT19; CD3D and CD79A; NKG7, KIT, and MKI67; DCN and PECAM1.

To characterize the cellular landscape and heterogeneity of LUAD, we performed single-cell transcriptomic analysis based on the GSE171145 dataset. After quality control and normalization, a unified Seurat object was constructed, and batch effects across samples were corrected using the Harmony algorithm. After quality control and normalization, a unified Seurat object was constructed, and batch effects across samples were corrected using the Harmony algorithm (**Supplementary Figures 1A, B**). Principal component analysis followed by shared nearest neighbor (SNN) graph-based clustering identified transcriptionally distinct cell clusters, which were visualized using UMAP (**Figure 1A**). **Figure 1B** displays the distribution of cells according to their patient of origin, indicating effective batch integration. Based on the expression of canonical marker genes, major cell types were annotated, including T cells, B cells, dendritic cells, macrophages, epithelial cells, endothelial cells, and fibroblasts (**Figure 1C**). Cellular composition varied across different stratifications (**Figure 1D**). Overall, T cells and epithelial cells were the most abundant populations. At the individual level (left), inter-patient variability in cell-type proportions was observed. Stratification by EGFR mutation subtype (middle) revealed subtype-specific differences in cellular composition. Notably, in the comparison by EGFR mutation status (right), wild-type LUAD samples exhibited higher infiltration of mast cells and fibroblasts, suggesting a potential influence of EGFR mutation on the non-immune stromal landscape. To validate the annotation, representative marker genes were visualized across the UMAP embedding (**Figure 1E**). For instance, EPCAM and KRT19 were enriched in epithelial cells, CD3D and CD79A marked T and B cells, while CD14 and CD68 were highly expressed in monocytes/macrophages, supporting the accuracy of cell-type classification. In addition, **Supplementary Figure 1C** provides a comprehensive overview of the expression patterns of key marker genes across major cell types and subclusters. It also integrates information on cell cycle phase, G2M scores, EGFR mutation status, and mitochondrial gene proportion, further supporting the accuracy of cell-type annotation and the stability of cellular states.

### 3.2 Differential cell–cell communication analysis between EGFR-mutant and wild-type LUAD

**Figure 2:**
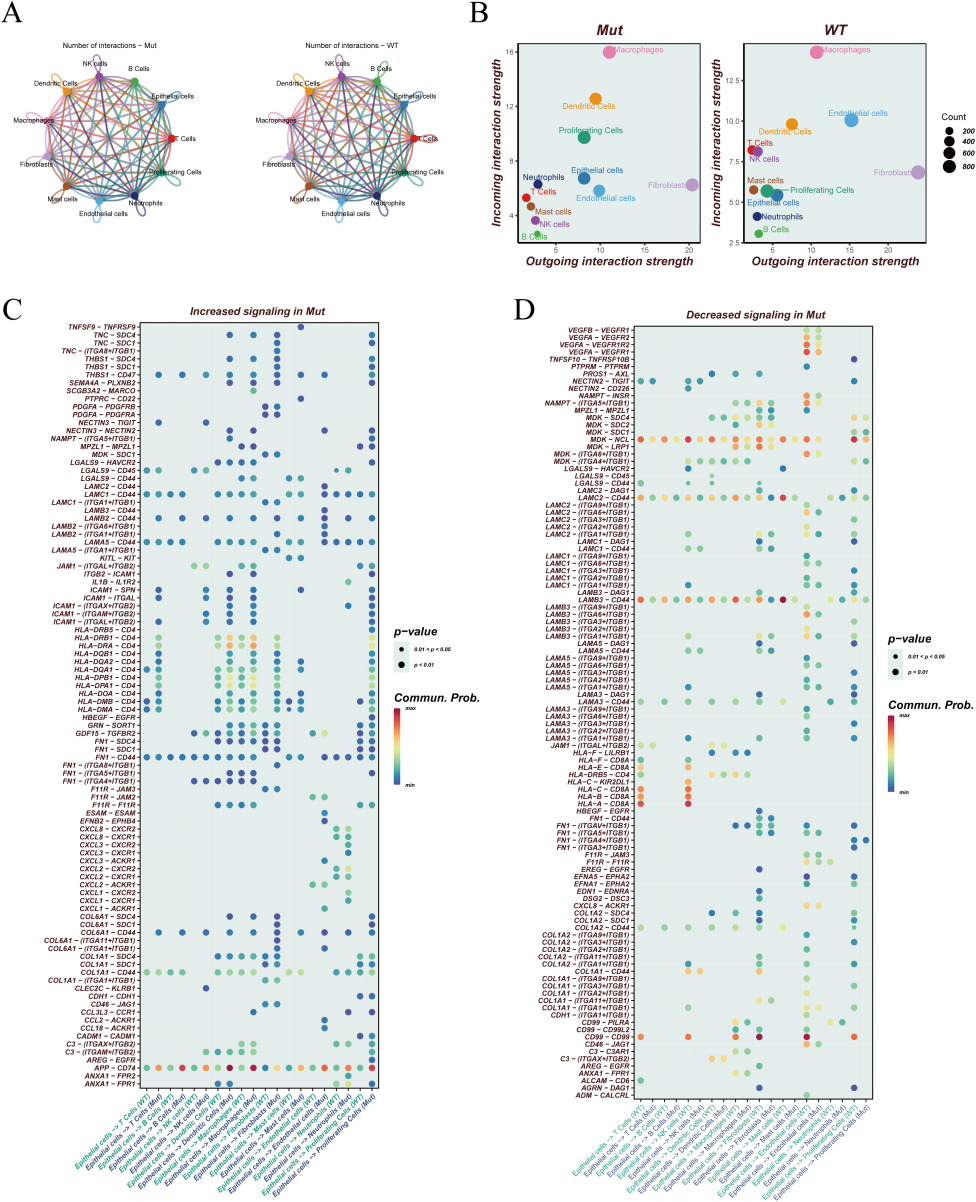
Differential cell–cell communication analysis between EGFR-mutant and wild-type LUAD. **(A)** Cell–cell communication networks in EGFR-mutant (Mut, left) and wild-type (WT, right) groups. Edge thickness indicates the strength of interaction between cell types. **(B)** Scatter plots displaying incoming (y-axis) and outgoing (x-axis) interaction strengths for each cell type in the Mut and WT groups. Bubble size reflects the number of interactions involving each cell population. **(C)** Heatmap showing signaling pathways with increased communication probability in the Mut group. Rows represent ligand–receptor pairs; columns represent sender–receiver cell pairs. Dot size indicates p-value significance, and color denotes communication probability. **(D)** Heatmap displaying signaling pathways with decreased communication activity in the Mut group, visualized in the same format as **(C)**.

To investigate how EGFR mutation status influences intercellular communication within the LUAD tumor microenvironment, cell–cell interaction networks were constructed separately for EGFR-mutant (Mut) and wild-type (WT) samples. **Figure 2A** provides a qualitative overview of the intercellular communication networks constructed separately for the EGFR-mutant (Mut) and wild-type (WT) groups. Nodes denote cell types, edge thickness encodes interaction strength, and node size reflects the participation of each cell type within the network. This panel is presented as an overview and is not intended for direct quantitative comparison between groups. **Figure 2B** illustrates the incoming and outgoing signaling strengths of each cell type in both groups. In the WT group, endothelial cells played a more prominent role in both sending and receiving signals, suggesting their functional importance in maintaining microenvironmental homeostasis. Across both groups, fibroblasts consistently served as the dominant signal senders, while macrophages were the major signal receivers, highlighting a conserved directionality in stromal–immune crosstalk. Pathways with significantly altered signaling activity in the mutant group were further identified. As shown in **Figure 2C**, a number of immune-related pathways exhibited increased activity in the EGFR-mutant group, including APP–CD74 and multiple HLA class II–CD4 ligand–receptor pairs (e.g., HLA-DPA1–CD4, HLA-DQA1–CD4, HLA-DRB1–CD4). These interactions are largely involved in antigen presentation and CD4^+^ T cell activation, indicating that EGFR mutation may impair antigen processing and helper T cell-mediated immune surveillance. In contrast, pathways with attenuated activity in the Mut group (**Figure 2D**) were primarily enriched in cell adhesion and extracellular matrix remodeling signals, such as LAMC2–CD44 and LAMB3–CD44. These interactions are associated with enhanced adhesion, migration, and invasive potential of tumor cells, suggesting that EGFR mutation may promote tumor progression and immune evasion by modulating specific adhesion-related communication axes.

### 3.3 Clustering analysis and identification of malignant epithelial cells

**Figure 3:**
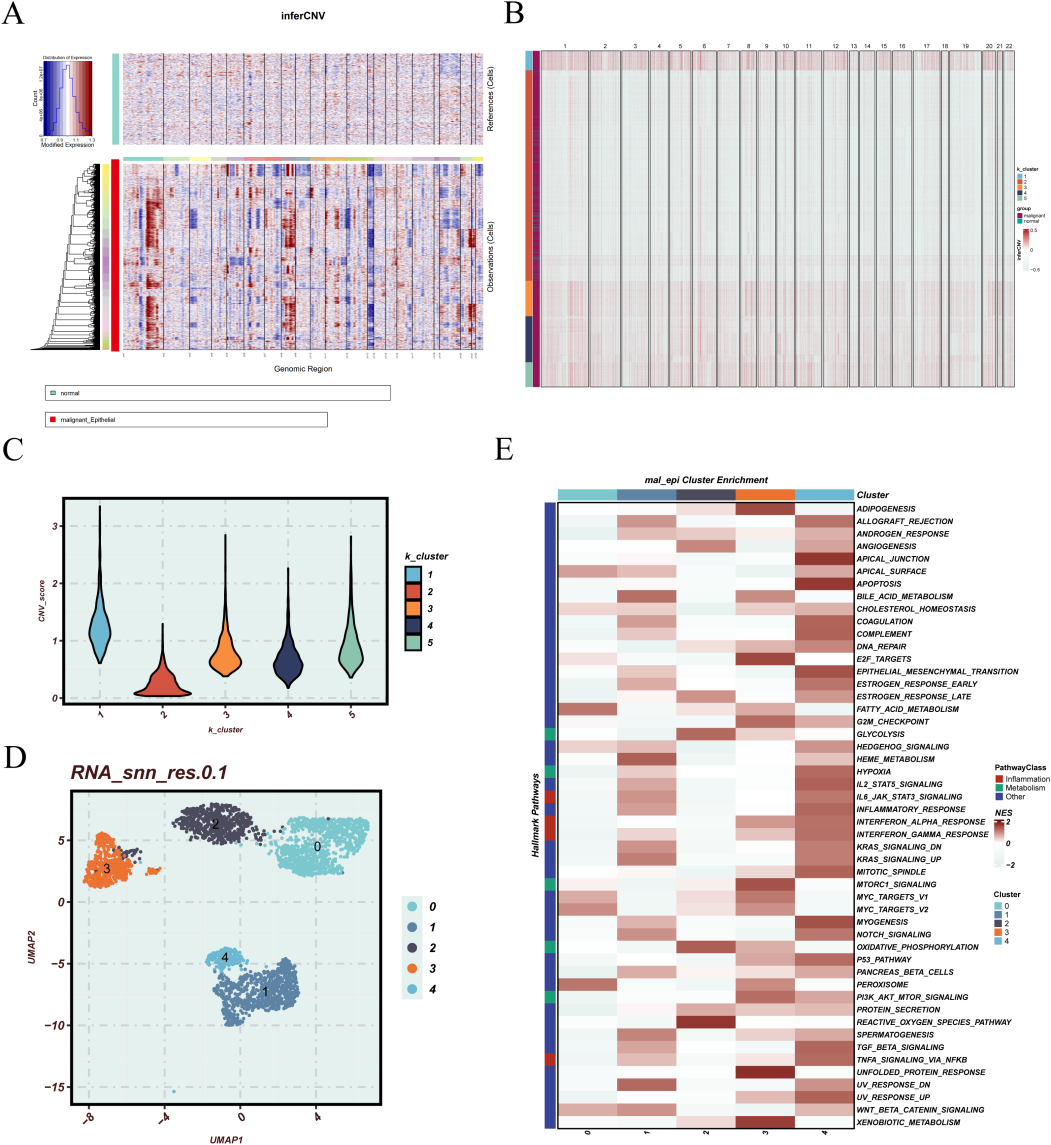
CNV inference, clustering, and functional characterization of malignant epithelial cells. **(A)** inferCNV plot illustrating the inferred copy number variation (CNV) profiles derived from single-cell RNA sequencing data. Normal epithelial cells are positioned at the top as the reference group, while epithelial cells under evaluation are displayed below. The heatmap color scale denotes relative chromosomal copy number changes, where blue represents regions of deletion and red represents regions of amplification. This arrangement facilitates visual comparison of CNV patterns between reference and test cells, enabling the identification of potential malignant phenotypes. **(B)** K-means clustering of all epithelial cells based on inferred CNV profiles, identifying distinct subpopulations with variable genomic alterations. **(C)** Violin plot comparing CNV scores across the five K-means clusters, reflecting differences in overall CNV burden. **(D)** UMAP plot of malignant epithelial cells extracted from EGFR-mutant patients, illustrating the spatial distribution of distinct epithelial subgroups. **(E)** Heatmap of pathway enrichment across malignant epithelial clusters, highlighting differential activity in biological processes.

CNV inference was performed on epithelial cells from LUAD samples to assess genomic instability. Using endothelial cells as a reference, the inferCNV heatmap revealed distinct CNV patterns, with widespread amplifications and deletions across various chromosomal regions in a subset of epithelial cells (**Figure 3A**). K-means clustering based on CNV profiles identified five epithelial subclusters with varying levels of genomic alterations (**Figure 3B**). Comparison of CNV scores showed that clusters 1, 3, 4, and 5 exhibited significantly higher CNV burdens compared to cluster 2 (**Figure 3C**), suggesting that these clusters likely represent malignant epithelial populations. Focusing on EGFR-mutant samples, malignant epithelial cells were extracted and visualized using UMAP, revealing distinct spatial separation among subclusters, indicative of pronounced intratumoral heterogeneity (**Figure 3D**). Functional enrichment analysis further demonstrated that these malignant subpopulations displayed diverse pathway activation patterns (**Figure 3E**), including epithelial–mesenchymal transition (EMT), cell cycle progression, DNA repair, apoptosis regulation, metabolism, and oxidative stress response, implying divergent functional roles in tumor progression. To evaluate the clinical significance of these malignant subpopulations, we calculated subcluster-specific signature scores in the TCGA cohort. Patients were stratified into high and low expression groups for survival analysis. As shown in **Supplementary Figure 2**, high scores for cluster_2 and cluster_3 were significantly associated with poorer survival outcomes (p < 0.001 and p = 0.005, respectively), while cluster_0 also demonstrated an unfavorable trend (p = 0.0031), highlighting their potential prognostic relevance.

### 3.4 Reconstruction of malignant epithelial cell differentiation trajectory

**Figure 4:**
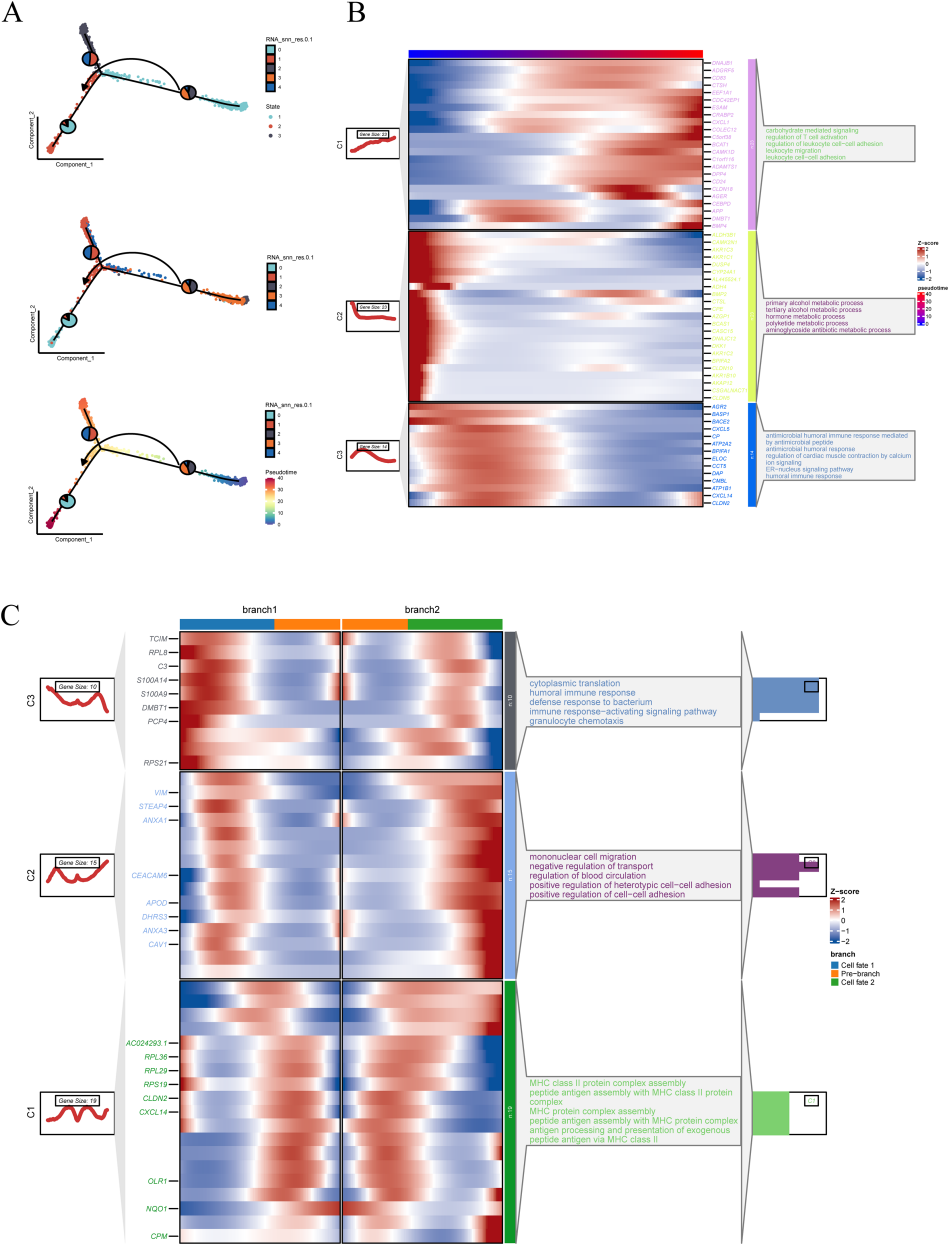
Pseudotime trajectory construction and branch analysis of malignant epithelial cells. **(A)** Pseudotime trajectory plot constructed using Monocle2, based on the top 2000 differentially expressed genes and reduced with the DDRTree algorithm. Cells are colored by state, RNA_snn_res.0.1 clusters, and pseudotime. **(B)** Heatmap of genes with expression changes along the pseudotime trajectory. The left panel shows gene clustering results, and the right panel presents functional annotations for each gene module. **(C)** Heatmap of branch-related genes identified through BEAM analysis. The two trajectory branches are shown with corresponding gene expression patterns and annotations.

To further investigate the transcriptional heterogeneity and differentiation dynamics of malignant epithelial cells, a pseudotime trajectory analysis was conducted. The top 2,000 differentially expressed genes across epithelial subclusters were selected to construct the trajectory using Monocle2, with dimensionality reduction performed via the DDRTree algorithm. Three distinct cell states and a continuous differentiation path were identified (**Figure 4A**). Notably, cluster 2 and 3 were primarily located at the early stage of the trajectory, while cluster 0, 1, and 4 were enriched at the terminal branches, suggesting a potential progression axis among malignant subpopulations. To characterize gene expression dynamics along the pseudotime axis, significantly varying genes were clustered and visualized in a heatmap (**Figure 4B**). Functional enrichment revealed that these gene modules were associated with biological processes such as regulation of leukocyte cell–cell adhesion, aminoglycoside antibiotic metabolic process, and humoral immune response, indicating immunological and metabolic reprogramming during progression. Branch-specific transcriptional programs were further dissected using BEAM analysis. Genes with significant expression divergence between branches were visualized in a branched heatmap and subjected to pathway enrichment analysis (**Figure 4C**). Branch 2 was enriched in terms including negative regulation of transport, positive regulation of heterotypic cell–cell adhesion, and mononuclear cell migration, while Branch 1 was predominantly associated with immune response–activating signaling pathways, suggesting distinct functional trajectories and potential cell fate bifurcations.

### 3.5 High-dimensional weighted gene co-expression network reveals functional modules in malignant epithelial cells

**Figure 5:**
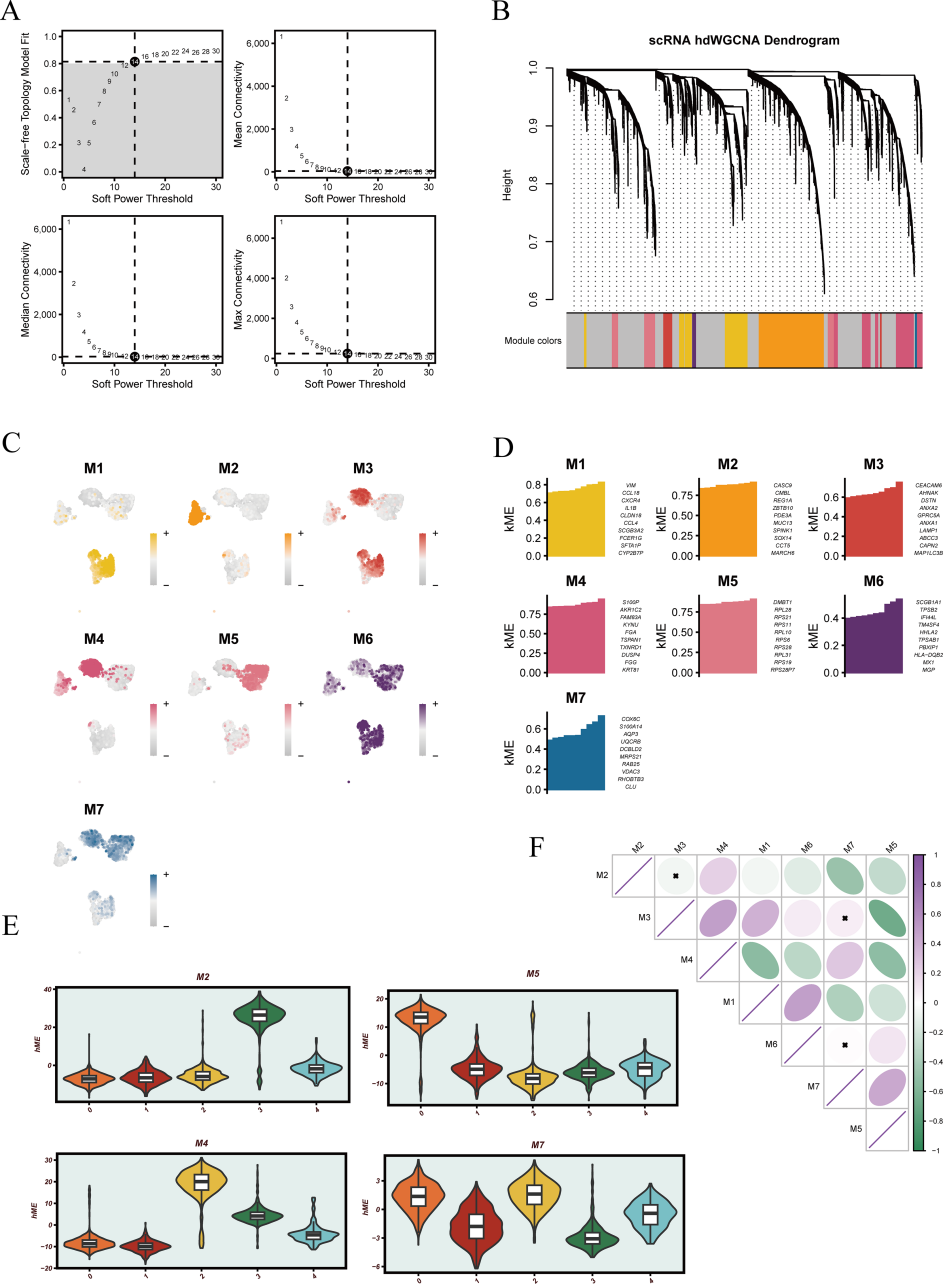
Overview of hdWGCNA workflow and visualization of gene co-expression modules. **(A)** Determination of the soft-thresholding power for network construction, based on scale-free topology fit and mean connectivity. **(B)** Gene clustering dendrogram generated from the weighted co-expression matrix, with identified modules represented by different colors. **(C)** UMAP projection showing module eigengene (ME) scores across different cell populations. **(D)** Bar plots displaying the top 10 genes with highest ME scores for each module. **(E)** Violin plots illustrating module eigengene score distributions across different groups. **(F)** Correlation matrix of module eigengenes among all identified modules.

To further elucidate the functional heterogeneity of malignant epithelial cells, a high-dimensional weighted gene co-expression network was constructed. The optimal soft-thresholding power was determined as 14 to ensure that the resulting network conformed to a scale-free topology (**Figure 5A**). Based on this threshold, multiple gene co-expression modules were identified, each represented by a distinct color (**Figure 5B**). UMAP projections of module-specific gene expression revealed distinct spatial distributions of each module within malignant cells (**Figure 5C**). The top 10 hub genes of each module were annotated (**Figure 5D**), providing a basis for downstream functional analyses. **Supplementary Figure 2** further visualizes the internal co-expression structure of each module, highlighting dense interactions among genes, particularly within modules M2, M4, M5, and M7, which showed strong intra-module connectivity.

Given that clusters 0, 2, and 3 were previously shown to be associated with patient prognosis, we focused on the modules enriched in these clusters. Violin plots revealed that module M2 was predominantly enriched in cluster 3, M4 in cluster 2, while M5 and M7 were highly expressed in both clusters 0 and 2 (**Figure 5E**), suggesting that these modules may regulate pathways related to poor prognosis. Correlation analysis among modules further revealed strong positive associations between M3 and M2, M3 and M7, and M6 and M7 (**Figure 5F**), indicating potential coordinated regulation and shared biological functions among these modules.

### 3.6 Machine learning-based generation of the EGFRmERS score for LUAD prognosis

**Figure 6:**
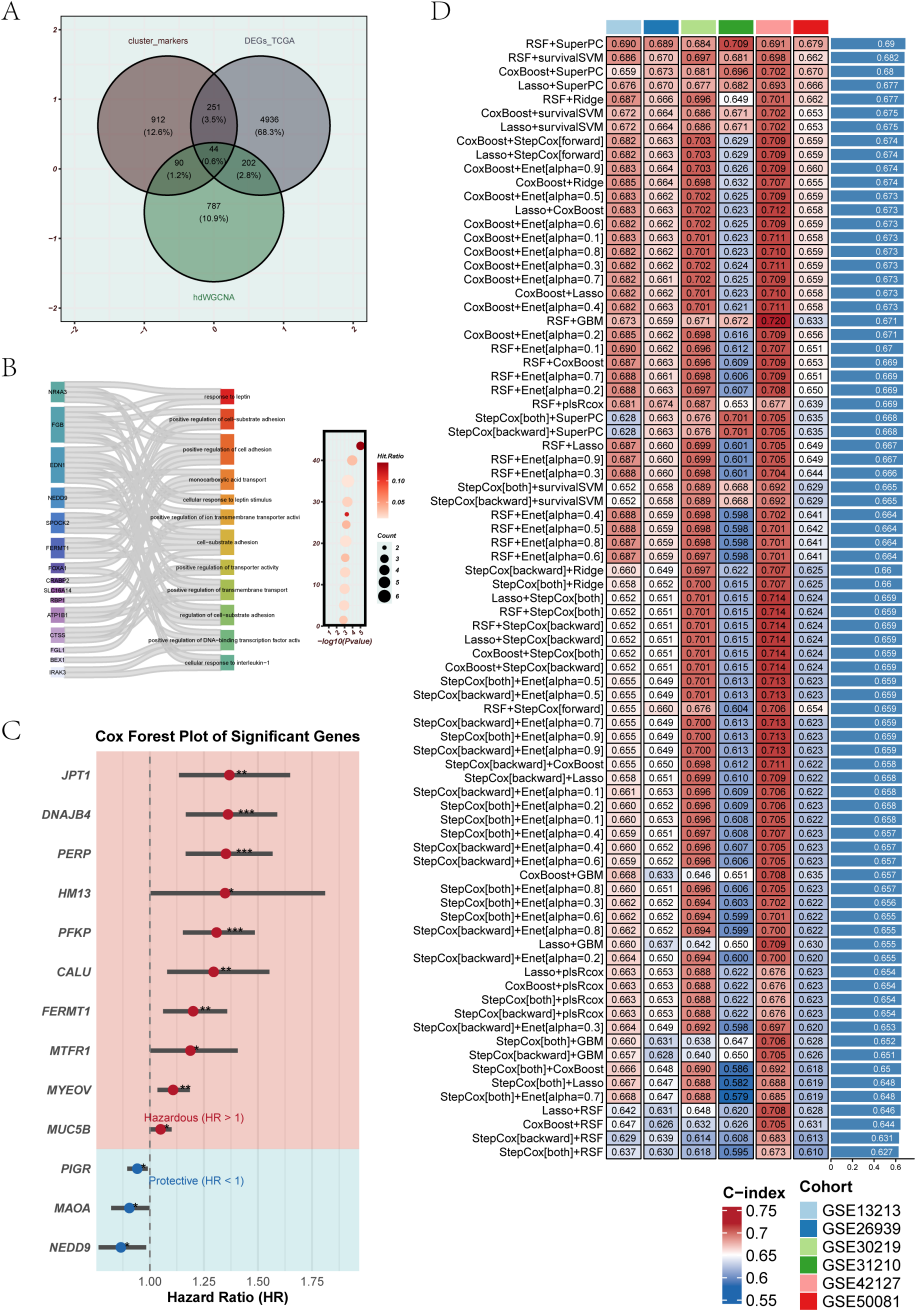
Integration of candidate genes and construction of prognostic models using machine learning. **(A)** Venn diagram showing the intersection of differentially expressed genes from TCGA, genes from hdWGCNA modules, and marker genes of epithelial subpopulations associated with prognosis. A total of 44 overlapping genes were identified. **(B)** GO enrichment analysis of the overlapping genes. The left Sankey plot illustrates the relationship between genes and biological pathways, while the right bubble plot indicates the significance and gene counts of enriched terms. **(C)** Univariate Cox regression analysis of the overlapping genes. Red represents risk genes (HR > 1), and blue indicates protective genes (HR < 1). **(D)** Multiple machine learning algorithms were applied to the 44 intersecting genes to construct prognostic models. Model performance was assessed using the C-index across six independent GEO validation cohorts, with deeper colors representing higher predictive accuracy.

To construct the EGFRmERS, we first intersected three sets of genes: differentially expressed genes from the TCGA cohort, hub module genes identified by hdWGCNA, and marker genes from malignant epithelial subclusters associated with prognosis. This integration yielded a set of candidate genes (**Figure 6A**). The detailed information of the intersecting genes can be found in **Supplementary Table 3**. Gene Ontology enrichment analysis revealed that these genes were primarily involved in immune-related processes, cell adhesion, and metabolic regulation, including representative pathways such as “response to leptin” and “positive regulation of cell–substrate adhesion” (**Figure 6B**). For GO enrichment analysis, genes without functional annotation or those unmapped in the GO database were excluded, and only the most significant and representative pathways with their corresponding genes were visualized; therefore, the number of genes displayed in **Figure 6B** is smaller than the total of 44 intersecting genes shown in **Figure 6A**. Subsequently, univariate Cox regression analysis was performed to screen for survival-associated genes (**Figure 6C**). Based on these genes, ten machine learning algorithms were employed to construct prognostic models, including stepwise Cox, Lasso, Ridge, partial least squares regression for Cox (plsRcox), CoxBoost, random survival forest (RSF), generalized boosted regression modeling (GBM), elastic net (Enet), supervised principal components (SuperPC), and survival support vector machine (survival-SVM). Ultimately, the optimal model was constructed by integrating RSF and SuperPC, forming the EGFRmERS scoring system (**Figure 6D**). The final EGFRmERS comprised nine genes: PERP, PFKP, DNAJB4, MYEOV, CALU, NEDD9, MTFR1, HM13, and PIGR.

**Figure 7:**
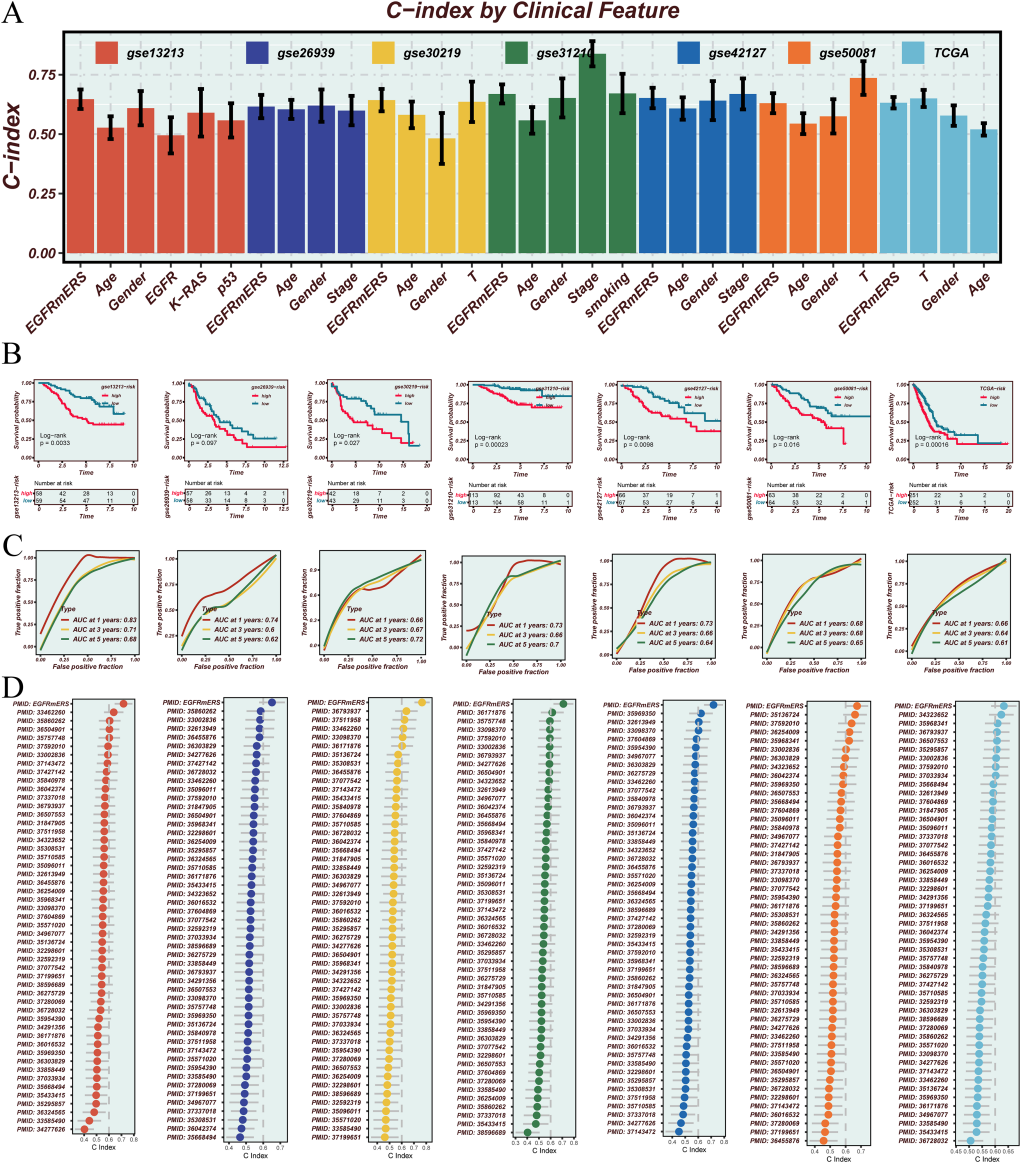
Validation and performance assessment of the EGFRmERS signature across multiple cohorts. **(A)** Comparison of the concordance index (C-index) between EGFRmERS and conventional clinical features (gender, age, stage) across seven independent cohorts. **(B)** Kaplan–Meier survival analysis based on median EGFRmERS scores to assess its prognostic stratification ability. **(C)** Time-dependent ROC curves evaluating the predictive accuracy of EGFRmERS for 1-, 3-, and 5-year overall survival. **(D)** Comparison of C-index values between EGFRmERS and previously published prognostic signatures for LUAD in each dataset.

The prognostic performance of EGFRmERS was evaluated in comparison with conventional clinical variables by calculating concordance index (C-index) across multiple datasets. Results showed that EGFRmERS exhibited consistently higher C-index values than traditional indicators such as age, gender, and TNM stage, indicating its superiority in individualized risk prediction (**Figure 7A**). Stratifying patients into high- and low-risk groups based on the median EGFRmERS score revealed significantly poorer survival outcomes in the high-score group across both the TCGA and external validation cohorts, as shown by Kaplan–Meier survival analyses (**Figure 7B**). Predictive accuracy for 1-, 3-, and 5-year overall survival was further supported by ROC analysis, with area under the curve (AUC) values exceeding 0.70 in all datasets (**Figure 7C**). Compared with previously reported LUAD prognostic models, EGFRmERS achieved the highest C-index values across all datasets examined, underscoring its robust and generalizable prognostic utility (**Figure 7D**). In **Figure 7D**, the predictive performance of our model and previous models in the TCGA-LUAD cohort was compared using C-index. The previously reported models were primarily linear prognostic models; we strictly calculated prognostic scores according to the gene lists and corresponding coefficients (or formulas) provided in the original publications, and then applied these scores to the TCGA-LUAD cohort for C-index evaluation.

### 3.7 Comprehensive evaluation of drug sensitivity and immunotherapy response based on EGFRmERS

**Figure 8:**
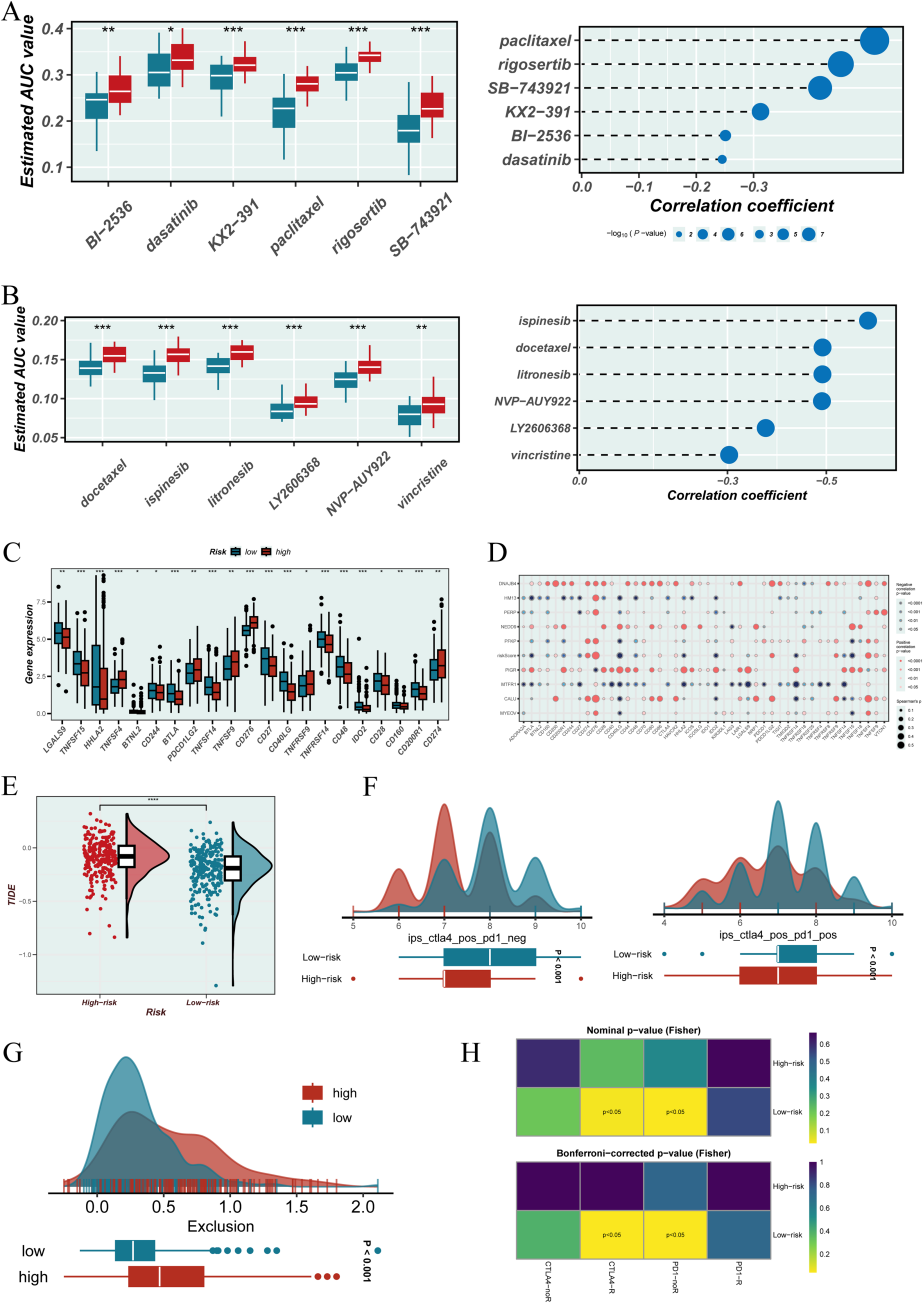
Evaluation of therapeutic relevance and immune landscape based on the EGFRmERS. **(A)** Drug sensitivity analysis using the CTRP database. Left: boxplots showing the estimated area under the dose–response curve (AUC) values for various compounds in the high and low EGFRmERS groups; lower AUC values indicate greater drug sensitivity. Right: Spearman correlation analysis between EGFRmERS scores and drug sensitivity profiles. **(B)** Drug sensitivity assessment from the PRISM database. Left: boxplots of AUC values for selected compounds in different EGFRmERS groups. Right: Spearman correlation coefficients reflecting the association between EGFRmERS scores and drug response. **(C)** Expression levels of immune checkpoint–related genes across EGFRmERS subgroups. **(D)** Correlation matrix showing associations between EGFRmERS scores, model genes, and immune checkpoint genes. **(E)** Comparison of TIDE scores between high and low EGFRmERS groups. **(F)** Distribution of immunophenoscore (IPS) components, including IPS-CTLA4 and IPS-PD1, across EGFRmERS subgroups. **(G)** Distribution of Exclusion scores from the TIDE platform in high versus low EGFRmERS groups. **(H)** SubMap analysis predicting differential response to immune checkpoint blockade therapies between the two EGFRmERS groups. * indicates P < 0.05, ** indicates P < 0.01, and *** indicates P < 0.001.

In the drug sensitivity analysis, samples with lower EGFRmERS scores exhibited increased sensitivity to multiple anticancer agents in both the CTRP and PRISM datasets, as reflected by lower AUC values (**Figures 8A, B**, left). Spearman correlation analysis further indicated a significant positive correlation between EGFRmERS scores and drug AUCs, suggesting that higher scores may be associated with increased drug resistance (**Figures 8A, B**, right). Analysis of immune checkpoint gene expression revealed that the high EGFRmERS group showed elevated expression of key immunoregulatory genes, including CD274 (PD-L1), TNFRSF9 (4-1BB), CD276 (B7-H3), and PDCD1LG2 (PD-L2) (**Figure 8C**). However, overall, the EGFRmERS score exhibited a negative correlation with most immune checkpoint-related genes (**Figure 8D**), implying a complex immunosuppressive profile in high-score patients. Further exploration showed that patients with high EGFRmERS scores had significantly attenuated TIDE scores (**Figure 8E**) and reduced IPS scores (**Figure 8F**), indicating a stronger immune evasion tendency and potentially diminished responsiveness to immune checkpoint blockade. In addition, the Exclusion score was also higher in the high-score group (**Figure 8G**), reflecting a more pronounced immunosuppressive microenvironment. Finally, SubMap analysis suggested that patients in the low EGFRmERS group were more likely to respond to anti–PD-1 therapy (**Figure 8H**), supporting the potential clinical utility of EGFRmERS in predicting immunotherapy responsiveness.

### 3.8 Evaluation of immune infiltration and stromal features based on EGFRmERS stratification

**Figure 9:**
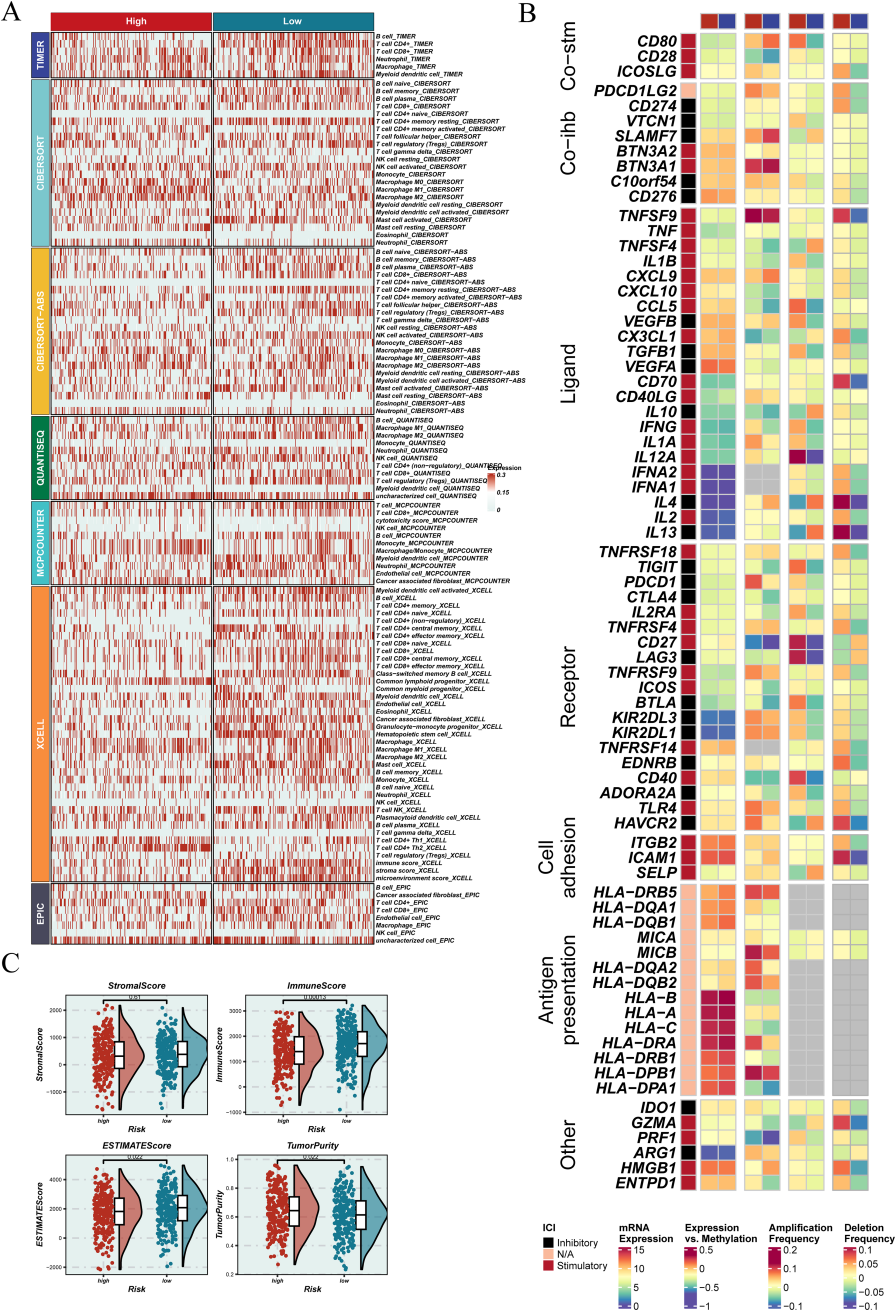
Association between EGFRmERS and immune microenvironment characteristics. **(A)** Heatmap illustrating the distribution of immune cell infiltration across different EGFRmERS groups, highlighting variations in the immune landscape between high- and low-score samples. **(B)** Overview of immune modulators—including co-stimulatory and co-inhibitory molecules, ligands, receptors, cell adhesion molecules, antigen-presenting markers, and other regulatory factors—showing their expression levels, mutation status, copy number alterations, and methylation profiles between high- and low-risk groups. **(C)** Comparative analysis of immune-related scores (StromalScore, ImmuneScore, ESTIMATE Score, and TumorPurity) between EGFRmERS groups, assessing their relationship with the tumor immune microenvironment.

Using seven immune infiltration methods from the TIMER2.0 database, the immune landscape of tumors with different EGFRmERS scores was evaluated (**Figure 9A**). While certain rare immune cell types showed minimal differences between groups, the majority of immune cell types—including CD8^+^ T cells, activated NK cells, and M1 macrophages—exhibited higher infiltration levels in the low-score group. This conclusion was further supported by additional immune metrics and functional analyses. The high-score group showed generally reduced immune cell infiltration, suggesting a more immunosuppressive microenvironment. Expression profiles of immunomodulatory genes also differed significantly between the two groups (**Figure 9B**). Additionally, ESTIMATE analysis revealed negative correlations between EGFRmERS scores and immune, stromal, and total scores (**Figure 9C**), supporting the notion that high EGFRmERS tumors are associated with a poorly infiltrated and stroma-deficient microenvironment.

### 3.9 Association of EGFRmERS with tumor mutational burden and mutation landscape

**Figure 10:**
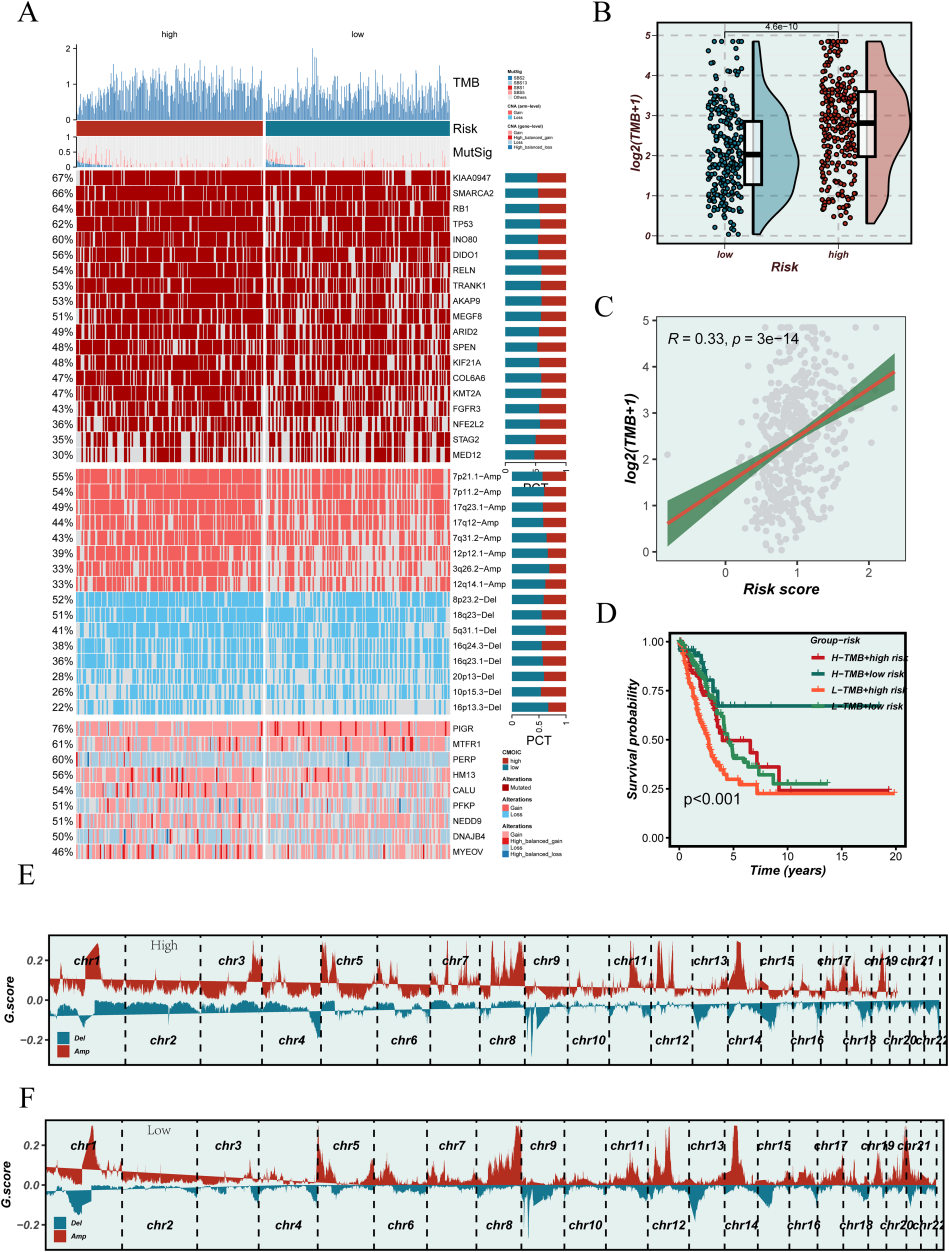
Association between the EGFRmERS score and genomic alterations. **(A)** Oncoplot illustrating the mutational landscape across high and low EGFRmERS subgroups, including tumor mutation burden (TMB), MutSig genes, copy number variations (CNVs), and frequently altered driver genes. **(B)** Comparison of TMB levels between high- and low-EGFRmERS groups. **(C)** Correlation analysis between EGFRmERS score and TMB. **(D)** Survival analysis stratified by TMB status and EGFRmERS subgroups. **(E)** Genome-wide copy number alteration profiles of the high EGFRmERS group based on GISTIC2.0 analysis. **(F)** Genome-wide copy number alteration profiles of the low EGFRmERS group based on GISTIC2.0 analysis.

To further investigate the potential association between the EGFRmERS score and genomic instability, we first examined the mutational landscape of the TCGA-LUAD cohort. **Figure 10A** summarizes the mutational landscape of the TCGA-LUAD cohort stratified by EGFRmERS, including mutation types, TMB annotations, and CNV profiles. This panel is intended as a qualitative overview rather than for direct quantitative comparison between groups. Comparison of TMB revealed significantly higher TMB levels in the high-score group (**Figure 10B**), and a positive correlation between EGFRmERS and TMB was identified (R = 0.33, p = 3e–14) (**Figure 10C**). Combined survival analysis indicated that patients with both high EGFRmERS and high TMB had the worst prognosis (**Figure 10D**). Furthermore, GISTIC2.0 analysis of CNVs demonstrated that the high-score group exhibited broader amplifications and deletions, particularly on chromosomes 3, 5, and 8 (**Figure 10E**), whereas CNV alterations were relatively limited in the low-score group (**Figure 10F**). These findings suggest that the EGFRmERS score may reflect distinct patterns of genomic instability.

### 3.10 Selection and functional validation of PERP as a key gene in LUAD progression

**Figure 11:**
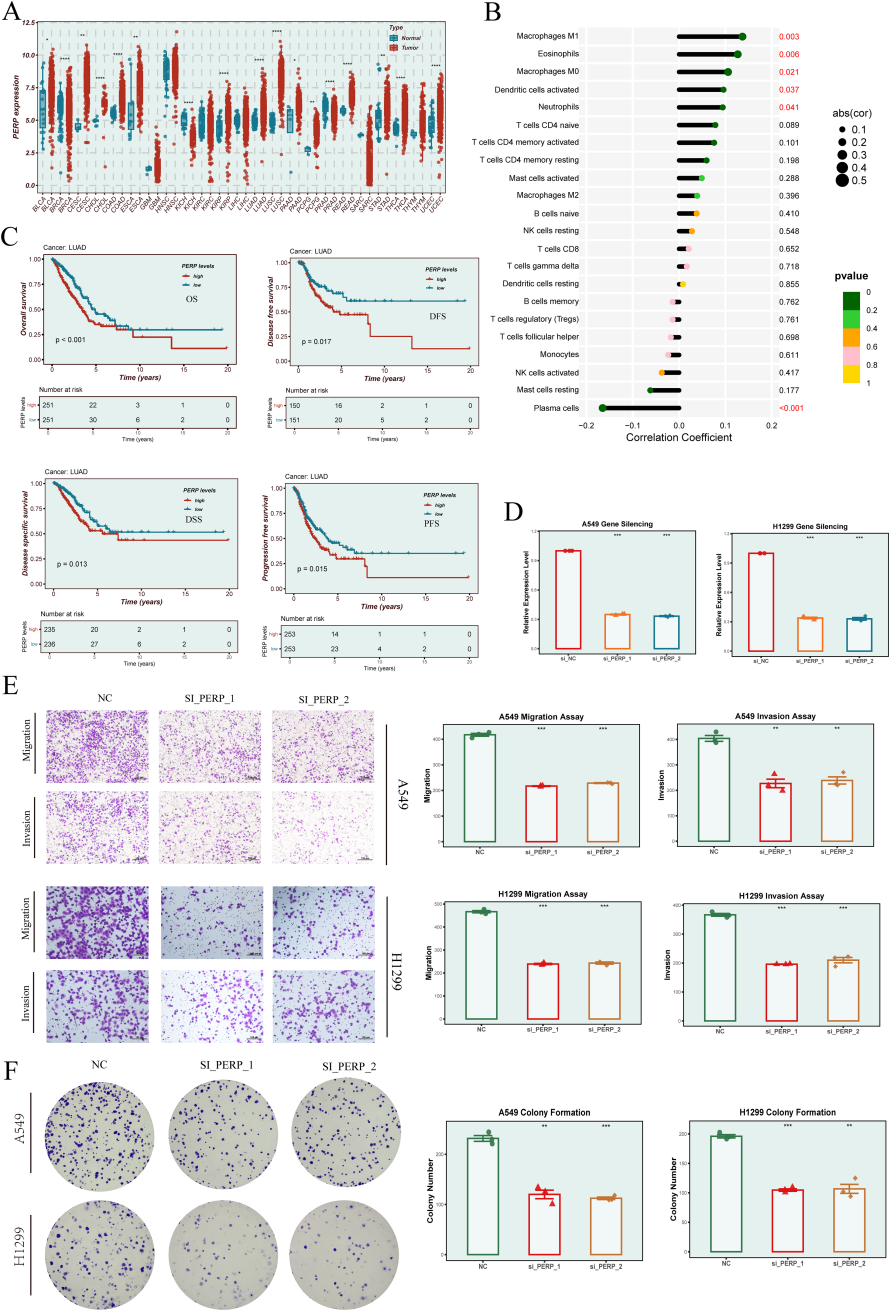
Expression profile, immune relevance, and functional validation of PERP. **(A)** Pan-cancer analysis of PERP expression across tumor and normal tissues. Red indicates tumor, blue indicates normal. **(B)** Correlation between PERP expression and immune cell infiltration estimated by the CIBERSORT algorithm, showing significant associations with multiple immune cell types. **(C)** Survival analyses (OS, DFS, DSS, and PFS) of PERP expression in the TCGA-LUAD cohort. **(D)** Silencing efficiency of PERP in A549 and H1299 cells following siRNA transfection. **(E)** Transwell migration and invasion assays evaluating the effects of PERP knockdown on A549 and H1299 cell motility. **(F)** Colony formation assays indicating enhanced proliferative capacity of PERP-silenced A549 and H1299 cells. * indicates P < 0.05, ** indicates P < 0.01, *** indicates P < 0.001 and **** indicates P < 0.0001.

Our EGFRmERS model comprised nine genes (PERP, PFKP, DNAJB4, MYEOV, CALU, NEDD9, MTFR1, HM13, and PIGR). As an initial step, we performed univariate Cox screening on candidate genes (**Figure 7C**), in which several genes—including JPT1, DNAJB4, and PERP—showed high hazard ratios. During subsequent model construction using Random Survival Forest (RSF) and Supervised Principal Components (SuperPC), feature selection removed JPT1 and certain other candidates; among the genes retained in the final model, PERP exhibited the highest hazard ratio (HR = 1.351). Considering its relatively high risk estimate within the final gene set, its elevated expression in LUAD and other malignancies, and literature support for its roles in cancer biology, PERP was prioritized for *in vitro* functional assays. We have clarified this selection process in the revised manuscript. Pan-cancer analysis revealed that PERP expression differed significantly between tumor and normal tissues across various cancer types (**Figure 11A**). Immune infiltration analysis using the CIBERSORT algorithm indicated that PERP expression was significantly correlated with multiple immune cell populations, particularly M1 macrophages, dendritic cells, and several T-cell subsets (**Figure 11B**). Survival analysis in the LUAD cohort showed that high PERP expression was associated with worse OS, DFS, DSS, and PFS outcomes (**Figure 11C**). To validate its functional role, we constructed PERP knockdown models in A549 and H1299 cell lines using siRNA. qRT-PCR confirmed efficient gene silencing (**Figure 11D**). Transwell assays demonstrated that PERP knockdown promoted both migratory and invasive capabilities in lung cancer cells (**Figure 11E**), while colony formation assays showed enhanced proliferative capacity following PERP silencing (**Figure 11F**).

## 4 Discussion

Lung cancer remains the leading cause of cancer-related deaths worldwide, imposing a significant public health burden due to its high incidence and mortality rates (32, 33). LUAD, the most common histological subtype of NSCLC, is driven by a complex interplay of genetic and microenvironmental factors, leading to marked heterogeneity in clinical outcomes among patients (34). Although the advent of targeted therapies and immune checkpoint inhibitors has improved survival for certain individuals, accurately identifying high-risk patients and predicting treatment response remains a critical challenge in clinical management (35, 36).

EGFR mutations are among the most pivotal molecular events in LUAD and have been widely applied in guiding targeted therapy (37). However, substantial heterogeneity exists within EGFR-mutant patients, as responses to TKI vary considerably, and resistance often emerges during treatment. Furthermore, many EGFR-mutant LUAD patients exhibit low responsiveness to immunotherapy, reflecting complex biological diversity within this molecular subtype (38, 39). These challenges underscore the need for refined risk stratification frameworks that incorporate additional molecular features to improve prognostic accuracy and therapeutic precision.

In this study, we focused on malignant epithelial cells within EGFR-mutant LUAD. By leveraging single-cell RNA sequencing data and integrating inferCNV, pseudotime trajectory, and co-expression network analyses, we characterized heterogeneity in malignancy and functional phenotypes across epithelial subpopulations, identifying specific cell groups associated with poor prognosis. Subsequently, we incorporated TCGA and multiple external validation cohorts to construct and evaluate a robust EGFRmERS. This model was developed using ten mainstream machine learning algorithms, integrating differentially expressed genes, single-cell markers, and prognostic features. EGFRmERS consistently outperformed conventional clinical parameters and previously published models across various datasets, highlighting its broad applicability across molecular backgrounds and populations.

Functional enrichment analyses revealed that high EGFRmERS scores were closely associated with pathways involved in cell adhesion, metabolic regulation, and immune suppression. Additionally, EGFRmERS positively correlated with several immune checkpoint molecules and immunotherapy response indicators such as TIDE and IPS scores, suggesting its potential utility in predicting immunotherapeutic sensitivity. Drug sensitivity analysis further indicated that patients with high EGFRmERS scores exhibited heightened sensitivity to a range of anti-tumor agents, offering guidance for personalized therapeutic selection.

Among the model genes, we focused on PERP due to its potential biological relevance. Previous studies have shown that PERP may act as a tumor suppressor by maintaining adhesion-dependent growth and promoting apoptosis, with well-established roles in melanoma and breast cancer (40–42). However, its function in lung cancer remains unclear, and some studies suggest that high PERP expression may be linked to poor prognosis. In our analysis, PERP was significantly upregulated in the high EGFRmERS group and positively associated with multiple immunosuppressive features, indicating that it may not only sustain malignant phenotypes within tumor cells but also contribute to immune evasion through tumor microenvironment remodeling. Follow-up qRT-PCR and functional assays further validated the oncogenic role of PERP in LUAD, supporting its potential as a therapeutic target.

Despite the systematic integration of single-cell and bulk transcriptomic data and the application of multiple machine learning algorithms with validation across independent cohorts, this study has several limitations. First, the model was developed using retrospective data from public databases; future validation in prospective clinical cohorts is necessary. Second, although the function of key genes such as PERP was confirmed *in vitro*, further studies using *in vivo* models and clinical specimens are needed to elucidate their mechanistic roles. Lastly, EGFRmERS performance may still be influenced by sample heterogeneity and the completeness of clinical annotations, necessitating future integration of multi-omics data to enhance model robustness.

In conclusion, this study constructed a novel EGFRmERS based on single-cell transcriptomic heterogeneity, machine learning algorithms, and multi-cohort validation. EGFRmERS not only effectively stratifies patient prognosis but also provides insights into immunotherapy response and drug sensitivity, offering a promising strategy for precision stratification and individualized therapy in EGFR-mutant LUAD.

## Data Availability

The original contributions presented in the study are included in the article/**Supplementary Material**. Further inquiries can be directed to the corresponding author.

## Supplementary material

Supplementary Figure 1 Batch correction and marker gene expression profiling in LUAD single-cell datasets. **(A, B)** Distribution of samples before and after Harmony batch correction, showing the effect of integration across datasets. **(C)** Dot plot showing the expression patterns of canonical marker genes across annotated main cell types (left panel) and transcriptional subclusters (right panel). The size of the dots represents the percentage of cells expressing each gene, and the color indicates the normalized average expression (z-score). The top annotations indicate the distribution of cell cycle phase (G1, S, G2M), G2M scores, EGFR mutation status (19del, L858R, wild-type), and mitochondrial gene percentage (pMT), providing additional context for cellular identity and state.

Supplementary Figure 2 Survival analysis of malignant epithelial subclusters. **(A–E)** Kaplan–Meier survival curves for cluster_0 to cluster_4 subpopulations based on subcluster-specific scores in TCGA samples. Patients were stratified into high and low expression groups, and overall survival differences were analyzed. Time (years) is shown on the x-axis, survival probability on the y-axis, with corresponding p-values and risk tables displayed. Note: For the subcluster-specific signature analyses shown in **Supplementary Figures 2A–E**, patients were dichotomized using the optimal cut-off determined by survminer::surv_cutpoint, rather than the median.

Supplementary Figure 3 Visualization of co-expression networks for the seven modules (M1–M7) identified through high-dimensional weighted gene co-expression network analysis (hdWGCNA) in malignant epithelial cells. Each node represents a gene, and edges represent co-expression relationships, with denser connections indicating stronger intra-module correlation.
