# Supplementary figures and images for: Integrative modeling of malignant epithelial programs in EGFR-mutant LUAD via single-cell transcriptomics and multi-algorithm machine learning
Source: Front Immunol. 2025 Oct 14;16:1661679. doi: 10.3389/fimmu.2025.1661679 (PMC12558860; doi:10.3389/fimmu.2025.1661679)

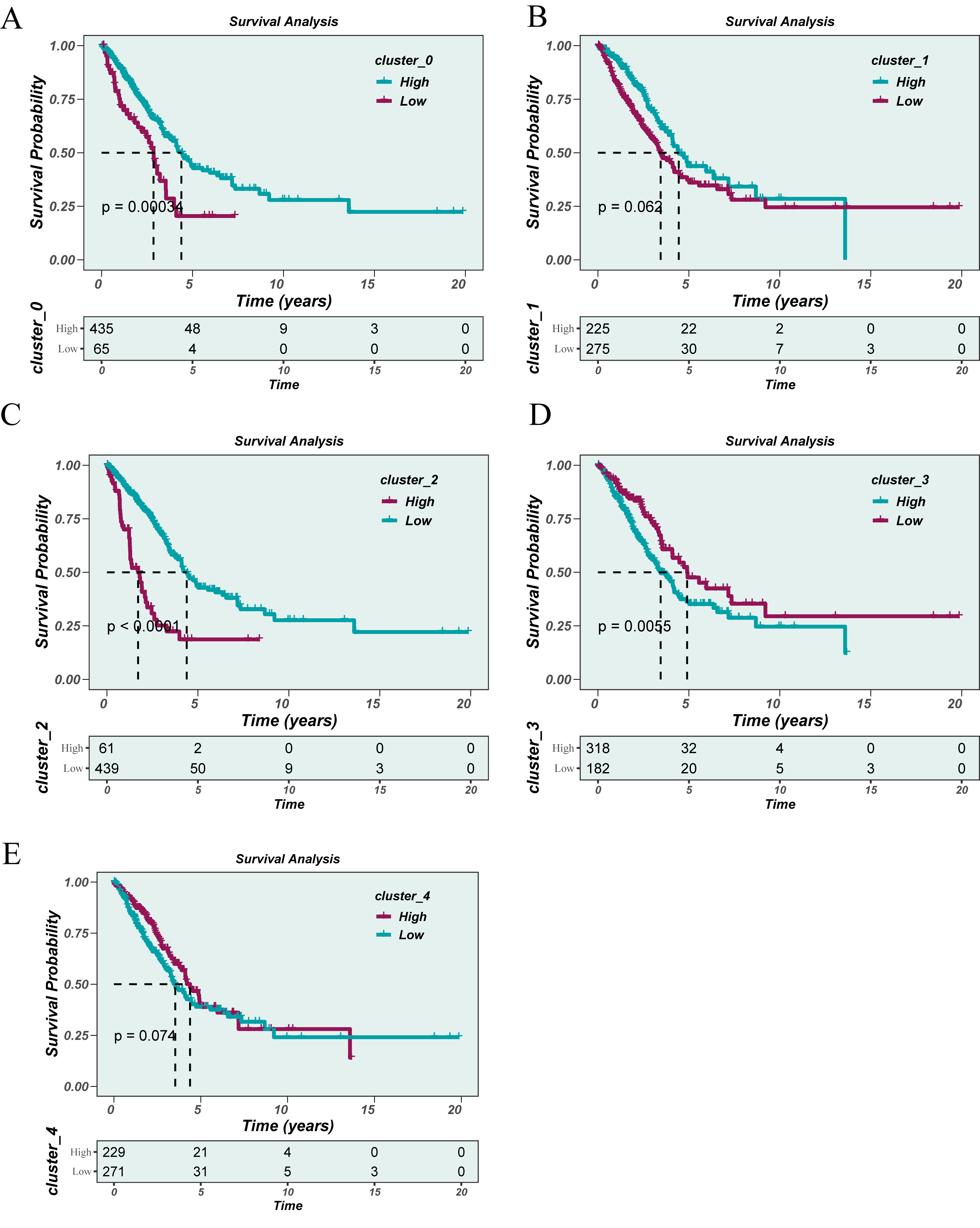

Supplement: Supplementary Figure 1 — Batch correction and marker gene expression profiling in LUAD single-cell datasets. (A, B) Distribution of samples before and after Harmony batch correction, showing the effect of integration across datasets. (C) Dot plot showing the expression patterns of canonical marker genes across annotated main cell types (left panel) and transcriptional subclusters (right panel). The size of the dots represents the percentage of cells expressing each gene, and the color indicates the normalized average expression (z-score). The top annotations indicate the distribution of cell cycle phase (G1, S, G2M), G2M scores, EGFR mutation status (19del, L858R, wild-type), and mitochondrial gene percentage (pMT), providing additional context for cellular identity and state. [file Image1.tif]

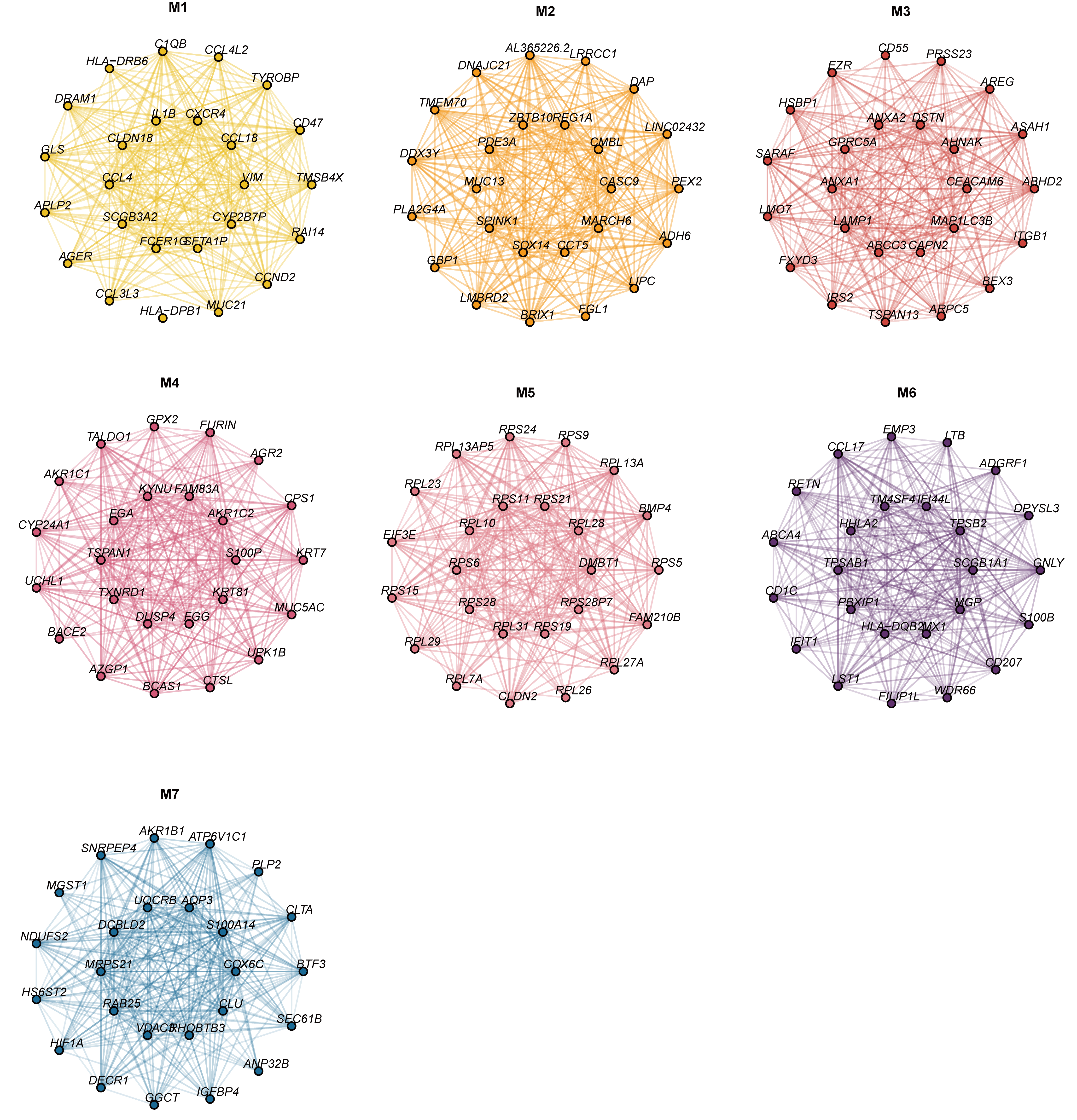

Supplement: Supplementary Figure 2 — Survival analysis of malignant epithelial subclusters. (A–E) Kaplan–Meier survival curves for cluster_0 to cluster_4 subpopulations based on subcluster-specific scores in TCGA samples. Patients were stratified into high and low expression groups, and overall survival differences were analyzed. Time (years) is shown on the x-axis, survival probability on the y-axis, with corresponding p-values and risk tables displayed. Note: For the subcluster-specific signature analyses shown in Supplementary Figures 2A–E , patients were dichotomized using the optimal cut-off determined by survminer::surv_cutpoint, rather than the median. [file Image2.tif]

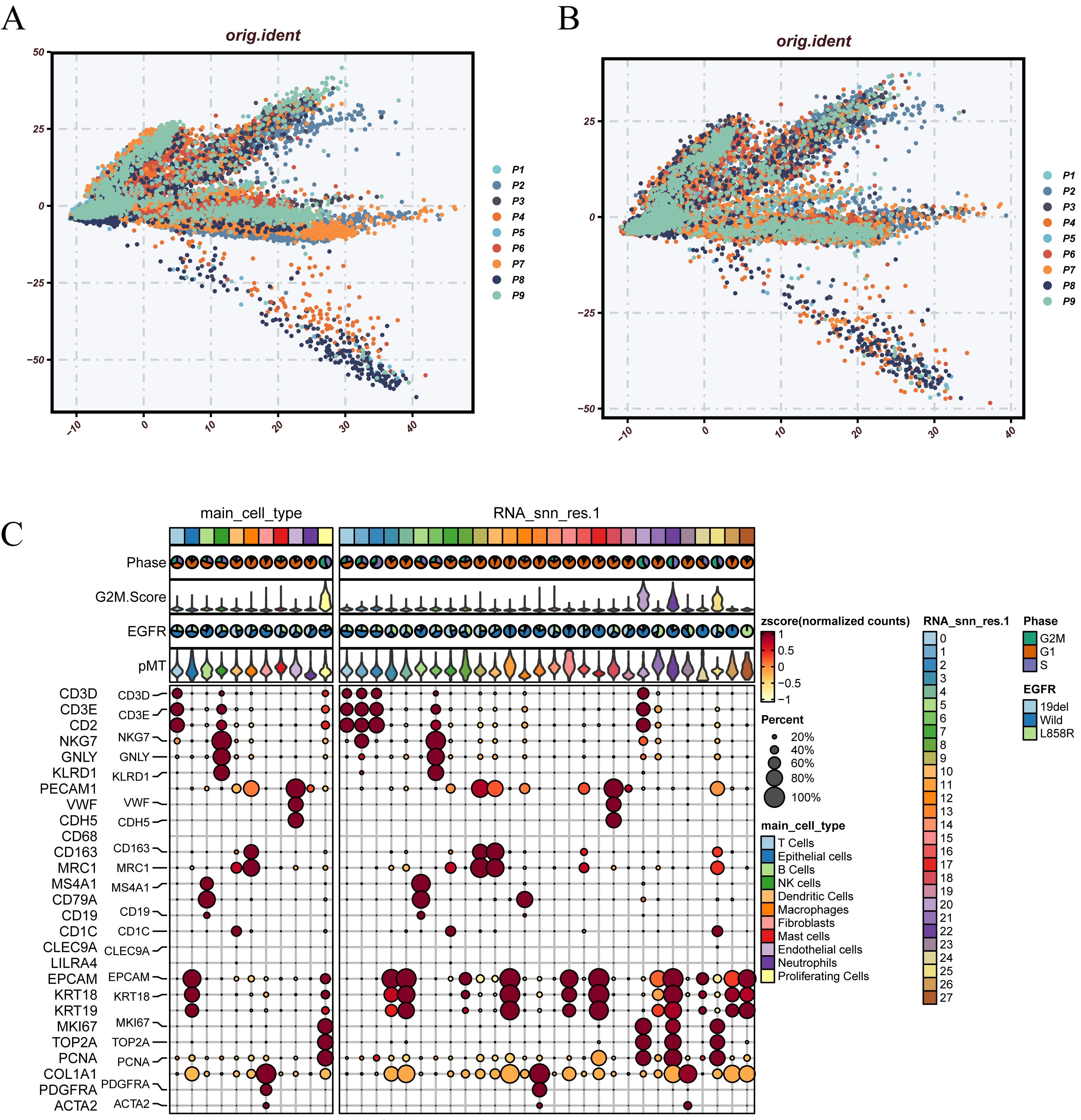

Supplement: Supplementary Figure 3 — Visualization of co-expression networks for the seven modules (M1–M7) identified through high-dimensional weighted gene co-expression network analysis (hdWGCNA) in malignant epithelial cells. Each node represents a gene, and edges represent co-expression relationships, with denser connections indicating stronger intra-module correlation. [file Image3.tif]
